# MEK/ERK addiction in CNL/aCML

**DOI:** 10.18632/oncotarget.22283

**Published:** 2017-11-03

**Authors:** Meenu Kesarwani, Zachary Kincaid, Mohammad Azam

**Affiliations:** Mohammad Azam: Division of Experimental Hematology and Division of Pathology, Cincinnati Children’s Hospital Medical Center, Cincinnati, Ohio, USA

**Keywords:** CNL, aCML, Trametinib, ERK1/2, KSR1

**Figure 1:**
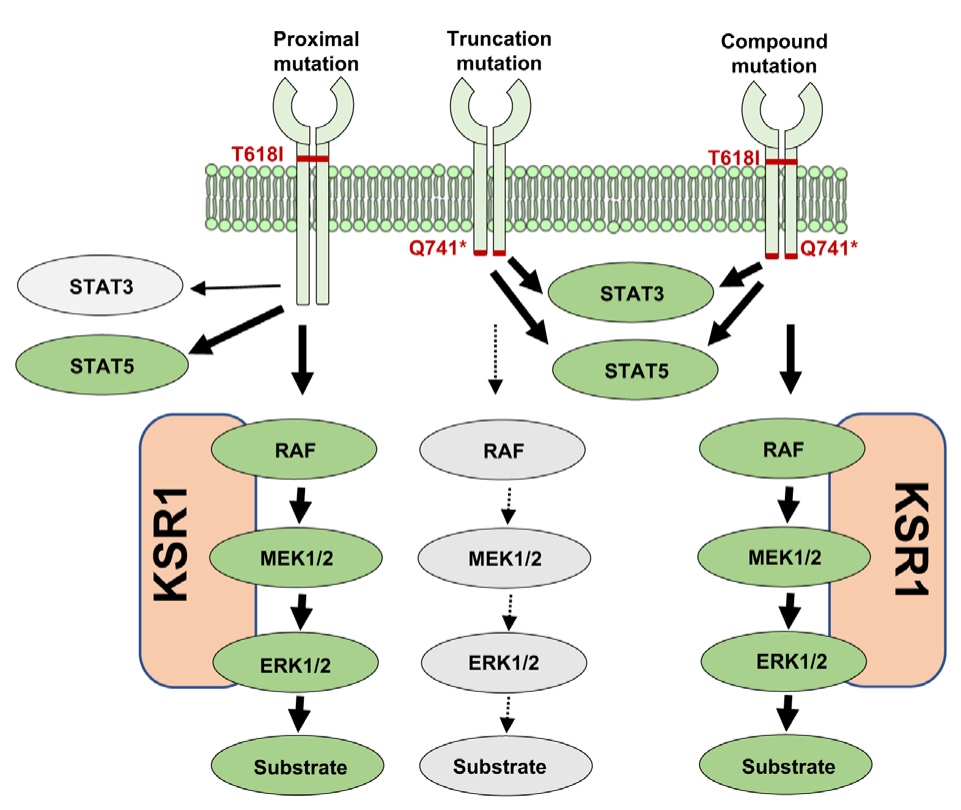
A Model for differential Signaling between leukemic and non-leukemic CSF3R mutations Non-leukemic truncation mutations in *CSF3R* constitutively activate both STAT3 and STAT5 in the absence of ligand. In contrast, membrane proximal mutations fail to activate STAT3, but fully activate STAT5, while compound mutations show full activation of both STAT3 and STAT5. Interestingly, both membrane proximal and compound mutations of CSF3R constitutively activate MAPK pathway by inducing the adaptor protein KSR1. In contrast, truncation mutations do not overexpress KSR1, therefore fail to activate MEK and ERK in the absence of ligand. Thus, CSF3R induced leukemia is dependent on enhanced MEK/ERK signaling.

Recent discovery of mutations in CSF3R and activation of JAK-STAT signaling in chronic neutrophilic leukemia (CNL) and atypical CML (aCML) provided the rationale for tyrosine kinase inhibitor (TKI) therapy targeting JAK2 [1]. Mutations in CSF3R are clustered in two groups (Figure 1). The first group encompasses missense mutations either in the membrane-proximal (T618I) or in the transmembrane domain (T640N) causing ligand independent activation of CSF3R. The second group of mutations is clustered in the cytoplasmic domain that causes premature termination of the receptor [1]. Membrane-proximal mutations alone are frequently found in CNL patients, in contrast, truncation mutations are less frequent and rarely found in solitude. Truncation mutations in CSF3R were initially reported from the patients of Severe Congenital Neutropenia (SCN) where it was implicated in leukemic progression. However, expression of truncated receptors alone in mice is not sufficient to induce leukemic transformation [2], thus, providing an explanation why truncation mutations alone are rarely found in CNL/aCML. In most cases, truncation mutations were reported to be identified with another proximal mutation (called compound-mutations). Given that the truncation mutations are non-leukemic, suggests that compound mutations could be either non-leukemic or have compromised leukemia. However, Maxson et.al. observed that proximal and truncation mutations displayed altered activation of downstream signaling resulting in differential sensitivity to tyrosine kinase inhibitors, ruxolitinib (proximal mutation) and dasatinib (truncation mutations) [1].

Patients harboring compound-mutations were presumed to be non-leukemic or have weakened leukemia, and most-likely to be sensitive to both inhibitors. In contrast to the previous finding, we observed that both proximal and truncation mutations are sensitive to ruxolitinib, but resistant to dasatinib. Moreover, *in vivo* mouse models show that the truncation mutations are not leukemogenic and are insensitive to dasatinib. Surprisingly, compound mutations induced aggressive leukemia and are resistant to both ruxolitinib and dasatinib treatments [3]. This observation provided evidence that truncation mutation by itself is non-leukemic but would exert synergistic response to proximal mutation resulting into therapy-resistant aggressive leukemia [3]. Ongoing clinical evaluation of ruxolitinib in CSF3R mutated CNL patients shows mixed response, where some patients show clinical benefits, while others are refractory to TKI treatment. This is in agreement with the *in vivo* data that supports the notion that secondary mutations (acquisition of CSF3R-truncation mutation or epigenetic mutations, such as SETBP and ASXL1) will be resistant to ruxolitinib. Besides, the timing of acquisition of CSF3R mutations is critical for TKI response [4]. For instance, early acquisition of CSF3R-proximal mutations in CNL patients exhibits superior response to TKI in comparison to patients who acquired CSF3R mutation at the later stage of disease development [4]. Nonetheless, these observations provide evidence that targeting JAK-STAT pathways will be ineffective in patients harboring secondary mutations.

Clinical efficacy of targeted therapy has been attributed to oncogene dependence [5]. To identify the signaling node supporting the dependence in CSF3R induced leukemia, we performed a comparative expression profiling of leukemic (CSF3R proximal and compound mutation) and non-leukemic (CSF3R truncation mutation) cells, which revealed enhanced expression of Mapk adaptor protein KSR1 in leukemic cells. Consequently, leukemic cells show higher MEK and ERK activation (Figure 1). Conversely, cells expressing wild-type CSF3R or truncation mutants failed to activate Mek and Erk due to reduced KSR1 expression (Figure 1). Even increased concentrations of GCSF failed to restore MEK/ERK activation in cells expressing truncated-CSF3R [3]. These observations provided evidence that CSF3R induced leukemia is fueled by enhanced MAPK signaling. Thus, MEK/ERK signaling confer oncogene dependence in CSF3R induced leukemia. This suggest that targeting MEK or ERK would be equally effective in both CSF3R proximal-mutant and compound mutants. As envisioned, trametinib treatment completely suppressed leukemia in both proximal and TKI resistant compound mutations. Perhaps more importantly, an aCML patient lacking CSF3R mutations showed durable disease response to MEK inhibition, thus supporting the notion that regardless of tumor initiating mutation, both CNL and aCML are addicted to enhanced MAPK signaling [6]. Therefore, targeting MEK/ERK in CNL/aCML merits clinical evaluation.

Given both JAK2 and MEK inhibitors are predominantly cytostatic (do not eradicate the leukemic clone), identifying drug combinations that harness ‘MEK dependence’ to induce cell death in leukemic cells might be more effective in targeting resistance and perhaps effect curative response. Because CSF3R leukemic mutations activate both Jak2 and Mek, a combination of both inhibitors might exert better therapeutic response. But, a combination of ruxolitinib and trametinib failed to show a better response than each inhibitor alone [3]. This suggests that concurrent targeting of both pathways will not have additional benefits, perhaps exploring alternative approaches may offer better therapeutic response.

In addition to KSR1, we identified overexpression of Pak6 and Bcl2l1 genes in cells expressing leukemic CSF3R. We anticipate that a combination comprising trametinib and Bh3 mimetics (antagonizes Bcl2) or Pak6 inhibitors might induce clonal selectivity. Alternatively, targeting KSR1 by a recently developed small molecule inhibitor alone or in combination with MEK or JAK inhibitors might be more effective in suppressing the resistance [7]. Perhaps more importantly, targeting c-FOS and DUSP1, which has recently been shown to be commonly overexpressed during leukemic transformation and regulates the TKI response [8], in a combination of TKI may exert curative response by selectively eradicating the leukemic clones.
